# Clinical outcomes and safety of continuous immunotherapy beyond progression in patients with extensive-stage small cell lung cancer: a retrospective real-world study

**DOI:** 10.3389/fimmu.2025.1681545

**Published:** 2026-02-06

**Authors:** Chengjun Wang, Tiantian Xuan, Yanan Wang, Chuang Yang, Wen Zhao, Rongyu Zhang, Xue Meng, Jisheng Li

**Affiliations:** 1 Department of Medical Oncology, Qilu Hospital, Cheeloo College of Medicine, Shandong University, Jinan, Shandong, China; 2 Department of Medical Oncology, Qilu Hospital (Qingdao), Cheeloo College of Medicine, Shandong University, Qingdao, Shandong, China; 3 Shandong Cancer Hospital and Institute, Shandong First Medical University and Shandong Academy of Medical Sciences, Jinan, Shandong, China; 4 Department of Radiation Oncology, Shandong Cancer Hospital and Institute, Shandong First Medical University and Shandong Academy of Medical Sciences, Jinan, Shandong, China

**Keywords:** antiangiogenesis, chemotherapy, immunotherapy, progression, small cell lung cancer

## Abstract

**Background:**

Immunochemotherapy has been approved as first-line treatment for extensive-stage small cell lung cancer. However, second-line treatment options and whether continuous immunotherapy will improve clinical outcome are still controversial. This multi-center retrospective study aimed to investigate the efficacy of continuous immunotherapy for the patients who suffered progression from first-line immunochemotherapy.

**Methods:**

We retrospectively reviewed the medical records of patients with extensive-stage small cell lung cancer treated with first-line immunochemotherapy in three major medical centers in Shandong Province. The patients enrolled achieved disease control during first-line immunochemotherapy but subsequently suffered disease progression.

**Results:**

From January 2020 to December 2024, a total of 354 patients treated with first-line immunochemotherapy were enrolled. The first-line progression free survival was 6.60 (95%CI: 6.28-6.92) months. A total of 206 patients were enrolled to compare the efficacy of second-line therapies, including chemotherapy alone (C, 40 cases), chemotherapy + anti-angiogenic therapy (C+A, 17 cases), immunochemotherapy (I+C, 122 cases), immunotherapy + anti-angiogenic therapy (I+A, 11 cases) and immunochemotherapy + anti-angiogenic therapy (I+C+A, 16 cases). Therein, I+C+A group obtained the longest second-line progression free survival of 4.60 (95%CI: 2.71-6.50) months. The second-line progression free survival of I+C group was also longer than that of the C group (3.50, 95%CI: 3.07-3.93 vs 2.33, 95%CI: 1.66-3.01). Regarding overall survival, I+A group achieved the longest overall survival of 22.00 (95%CI: 11.39-32.61) months compared with 19.53 (95%CI: 16.81-22.26) months for I+C group. However, there were no statistical differences in second-line progression free survival and overall survival among the groups. In terms of safety, the rates of adverse events in the I+C and C groups were not statistically significant.

**Conclusions:**

Continuous immunotherapy beyond progression in extensive-stage small cell lung cancer shows the trend of prolonging second-line progression free survival, but does not improve the overall survival. Additionally, in the second-line treatment, chemotherapy remains an important cornerstone therapy and anti-angiogenic agent containing strategy may potentially improve survival.

## Introduction

Lung cancer was the most frequently diagnosed cancer in 2022, accounting for nearly 2.5 million new cases, which was equivalent to about one out of every eight cancers diagnosed globally, making up 12.4% of all cancer cases around the world. Additionally, lung cancer was the primary cause of cancer-related deaths, with an approximate 1.8 million fatalities, representing 18.7% of all cancer deaths (1). Small-cell lung cancer (SCLC) is a high-grade neuroendocrine carcinoma arising predominantly in current or former smokers and has an exceptionally poor prognosis (2). The Veterans Administration Lung Cancer Study Group (VALSG) staging system is widely used in both designing clinical trials and presenting data of SCLC, as it effectively distinguishes patients treated primarily with chemoradiotherapy (limited-stage disease) from those treated with systemic chemotherapy or chemoimmunotherapy (extensive-stage disease). At the time of diagnosis, approximately two-thirds of all cases of SCLC would present with extensive-stage disease (ES-SCLC) (3).

Until 2019, the combination of etoposide and platinum had been the standard treatment for ES-SCLC (4, 5). However, in 2019, evidence showed that adding anti-programmed death-ligand 1 (anti-PD-L1) immunotherapies such as atezolizumab (6, 7) and durvalumab (3, 8) to etoposide/platinum chemotherapy could improve survival, which were recommended as first-line therapeutic options for ES-SCLC. Then several immune-checkpoint inhibitors (ICIs) such as pembrolizumab (9), ipilimumab (10), tremelimumab (8), adebrelimab (11), serplulimab (12), tislelizumab (13) and toripalimab (14) were also investigated in the first-line treatment of ES-SCLC. And also, anti-angiogenic agents including bevacizumab (15–17) and anlotinib (18) were also explored for combination immunochemotherapy, indicating positive synergistic effects. Nevertheless, even though immunochemotherapy, with or without anti-angiogenic therapy, has demonstrated potential by providing an overall survival (OS) benefit of 2 to 4 months for patients with ES-SCLC when compared to chemotherapy alone, the goal of enhancing survival still remains an unfulfilled need. Moreover, progression after previous anti-Programmed Cell Death-1/Programmed Cell Death Ligand-1 (anti-PD-1/PD-L1) inhibitors is inevitable, and if continuous immunotherapy leads to more favorable prognosis were still undefined.

After progression during chemotherapy combined with immunotherapy, a potential approach to address drug resistance is to modify the type of chemotherapy drug, aiming to restore the responsiveness to immunotherapy and enable its continued effectiveness. However, most previous studies have mainly focused on the rechallenge of immunotherapy, neglecting the impact of continuous immunotherapy after progression. According to a subgroup study of Checkmate 025, continuing immunotherapy beyond progression has reduced tumor burden by ≥ 30% in patients with advanced or metastatic clear-cell renal-cell carcinoma (19). OAK study also demonstrated the survival benefit of continued atezolizumab after progression for advanced non-small cell lung cancer (NSCLC) patients (20). To the best of our knowledge, there are limited data on whether continuous ICIs beyond progression can be adopted in SCLC. This study aimed to investigate the role of continuous immunotherapy and addition of anti-angiogenic agents beyond progression after first-line immunochemotherapy in ES-SCLC patients, identifying the optimal treatment strategy and specific subgroups that may derive greater benefits from this strategy.

## Methods

### Patients

We retrospectively collected patients with ES-SCLC that received ICIs plus chemotherapy as first-line therapy from January 2020 to December 2024 in the Department of Medical Oncology of Qilu Hospital of Shandong University (Jinan, Shandong), the Department of Medical Oncology of Qilu Hospital of Shandong Province (Qingdao, Shandong), and Shandong Cancer Hospital. Given that the study is retrospective, there is no need to provide written informed consent. Our multicenter and retrospective study was carried out in compliance with the amended Declaration of Helsinki and got approval from the appropriate ethical committees.

### Inclusion and exclusion criteria

The main inclusion criteria were as follows: 1) patients with pathologically or histologically diagnosed ES-SCLC; 2) patients who received at least two cycles of anti-PD-1 or anti-PD-L1 agents in combination with chemotherapy during the first-line therapy; 3) at least one measurable lesion. The primary exclusion criteria are listed below: 1) limited-stage small cell lung cancer; 2) patients who did not receive anti-PD-1 or anti-PD-L1 agents during the first-line therapy; 3) no measurable lesions; 4) incomplete medical data; 5) exclusion due to other diseases or causes.

### Data collection

The basic information of the patients before treatment was collected, including age, gender, Eastern Cooperative Oncology Group performance status (ECOG PS), smoking status, etc. Clinical characteristics included the date of diagnosis, location of metastasis, as well as the specific medications used during first-line treatment, the number of treatment cycles, the mode of second-line treatment, and follow up indicators including efficacy evaluation, follow-up time, progression-free survival (PFS), OS, adverse events (AEs), and immune-related adverse events (irAEs) during the entire treatment period. Continuous immunotherapy beyond progression refers to class continuation allowing ICI agents different from those used in the first-line therapy or continuation of the same ICI agent. Patient information was retrieved and summarized from the hospital medical records.

### Endpoints

The primary endpoints included first-line PFS, second-line PFS, and OS. First-line PFS is defined as the time from the start of first-line therapy to progression. Second-line PFS is defined as the duration from the start of second-line therapy to the date of disease progression or death due to any cause. OS is defined as the duration from the date of treatment initiation to the date of death due to any cause. Radiological data were used to assess tumor response based on the Response Evaluation Criteria in Solid Tumors (RECIST) version 1.1, classifying tumor response to treatment as complete response (CR), partial response (PR), stable disease (SD), and progressive disease (PD). CR refers to the disappearance of all target lesions; PR indicates that the sum of the diameters of target lesions has decreased by more than 30% from the baseline; PD refers to an increase of 20% or more in the sum of the diameters of target lesions compared to the smallest recorded value, with an absolute increase of at least 5 millimeters, or the appearance of new lesions; SD means that the change in the sum of the diameters of target lesions that falls within the range of a 30% decrease to a 20% increase. The objective response rate (ORR) is defined as the proportion of patients achieving CR or PR. All patients were actively followed up until May 1, 2025, and follow-up information was obtained via phone or directly from electronic medical record system files. AEs and irAEs were assessed and graded according to the scores defined the National Cancer Institute Common Terminology Criteria for Adverse Events version 5.0 (CTCAE v5.0).

### Statistical analysis

We stratified the included patients based on age, gender, ECOG PS, smoking status, location of metastatic lesions, and treatment modality for baseline characteristics and analyzed the differences between baseline characteristics using Chi-square test and Fisher’s exact test. The best response status during first-line treatment was statistically analyzed. Kaplan-Meier method was employed to evaluate the PFS during first-line and second-line therapy as well as OS. The log-rank test was used to compare the differences in survival curves between groups, and the corresponding 95% confidence interval (95%CI) was calculated. All tests yielded two-sided P values, and those with a value <0.05 were regarded as statistically significant. Statistical analysis was performed using SPSS software (version 26.0), the graphs were generated using R software (version 4.4.1) and GraphPad Prism software (version 9.0).

## Results

### Patient clinical characteristics

**Figure 1:**
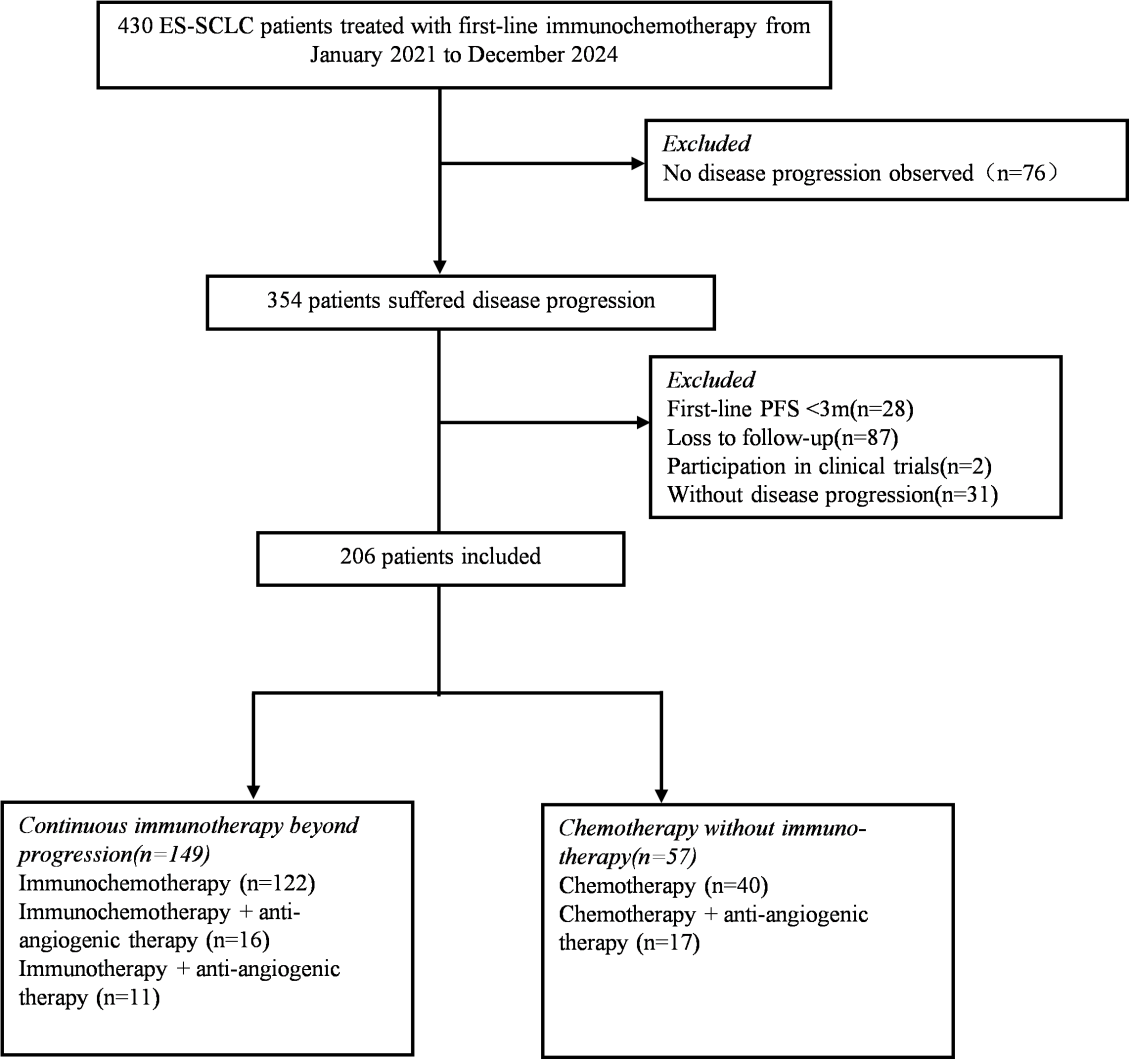
Flowchart of study design and patient screening. Study process. ES-SCLC, extensive-stage small-cell lung cancer. PFS, progression-free survival.

From January 2020 to December 2024, a total of 354 ES-SCLC patients treated with first-line immunochemotherapy who suffered disease progression were enrolled (**Figure 1**). The median age of the cases was 62 years (range, 36–84 years) and most patients had a performance status score of 0-1. There were 303 males (85.6%) and 230 (65.0%) with a history of smoking. As for metastatic sites, 96 (27.1%) cases presented with brain metastases, 125 (35.3%) with liver metastases, 123 (34.7%) with bone metastases. Among these, 241 (68.1%) patients received anti-PD-L1 antibody including atezolizumab, durvalumab, adebrelimab and envafolimab, 113 (31.9%) received anti-PD-1 antibody including serplulimab and others (**Table 1**). Pleural effusion was present in 145 patients (41.0%), and 24.9% of patients received locoregional thoracic radiotherapy. A minority of patients had the comorbidity of hypertension, diabetes, and coronary heart disease.

**Table 1:** Baseline clinical characteristics of the 354 patients.

Characteristics		No. (%)
Gender	Male	303 (85.6%)
	Female	51 (14.4%)
Age, years	Median age (range)	62 (36-84)
	<65	215 (60.7%)
	≥65	139 (39.3%)
ECOG PS	0	143 (40.4%)
	1	199 (56.2%)
	2	12 (3.4%)
Smoking history	No	124 (35.0%)
	Yes	230 (65.0%)
Metastatic sites	Liver	125 (35.3%)
	Bone	123 (34.7%)
	Brain	96 (27.1%)
Cycles of Immunotherapy	Median cycles(range)	6 (2-24)
Chemotherapy regimen	EC	231 (65.3%)
	EP	112 (31.6%)
	Others	11 (3.1%)
Immunotherapy regimen	Anti-PD-L1 antibody	241 (68.1%)
	Anti-PD-1 antibody	113 (31.9%)
Immunotherapy regimen	Durvalumab	97 (27.4%)
	Atezolizumab	77 (21.8%)
	Adebrelimab	61 (17.2%)
	Serplulimab	77 (21.8%)
	Others	42 (11.9%)
Pleural effusion	No	209 (59.0%)
	Yes	145 (41.0%)
Locoregional thoracic radiotherapy	No	266 (75.1%)
	Yes	88 (24.9%)
Complications	Hypertension	96 (27.1%)
	Diabetes	62 (17.5%)
	Coronary heart disease	27 (7.6%)

No., number; ECOG PS, Eastern Cooperative Oncology Group Performance Status; EC, etoposide combined with carboplatin; EP, etoposide combined with cisplatin; PD-L1, programmed cell death ligand 1; PD-1, programmed cell death 1.

### Efficacy of first-line therapy

**Figure 2:**
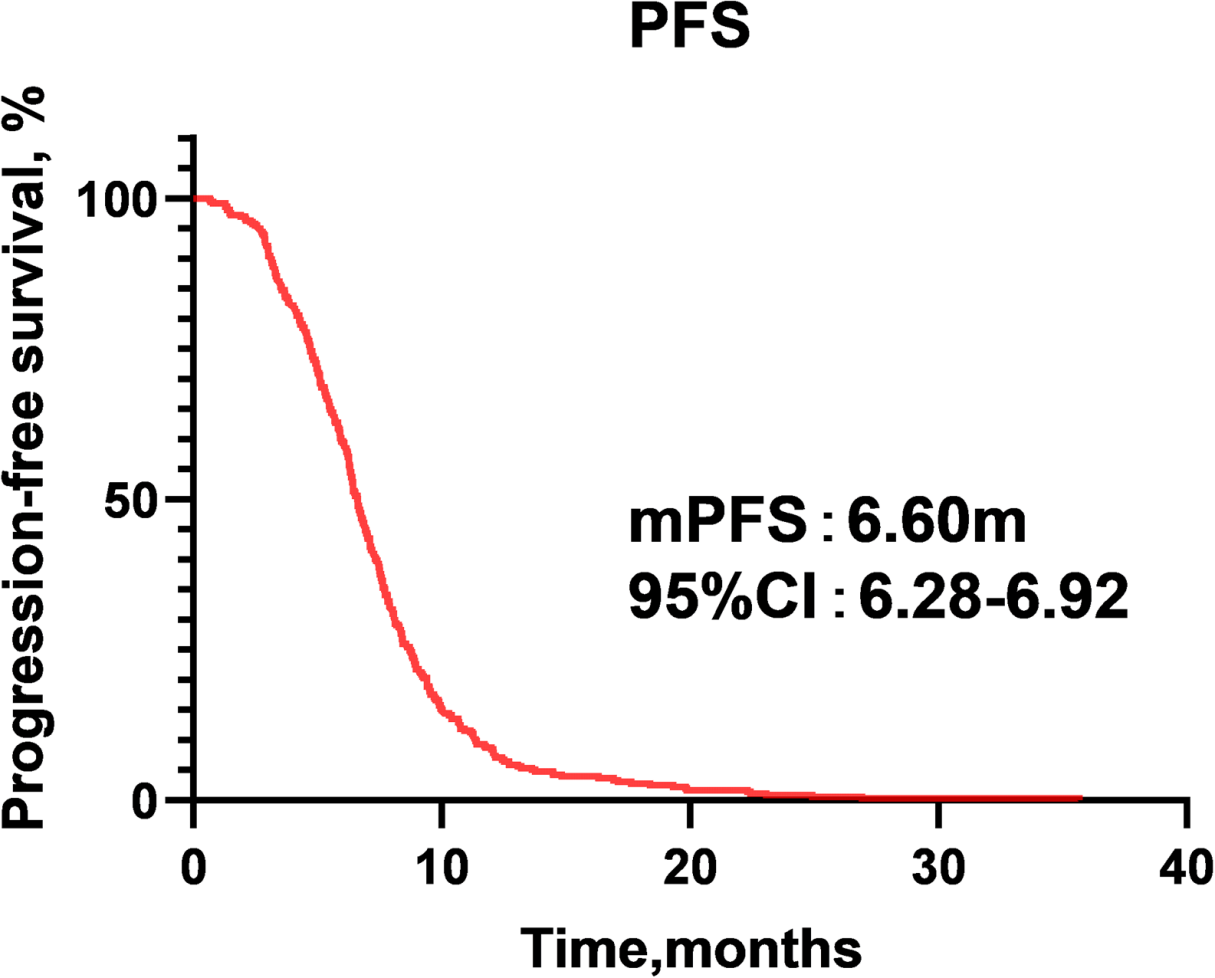
Kaplan-Meier analysis of PFS in patients treated with immunochemotherapy as first-line therapy. PFS, progression-free survival of first-line treatment; mPFS, median progression-free survival; m, months; CI, confidence interval.

Among 354 patients who received first-line immunochemotherapy and suffered disease progression, 3 cases achieved CR, PR was accomplished in 218 cases, and SD was observed in 126 cases. The median PFS (mPFS) of first-line therapy was 6.60 (95%CI: 6.28-6.92) months (**Supplementary Table 1**, **Figure 2**).

### Subgroup analysis of PFS during first-line therapy

**Figure 3:**
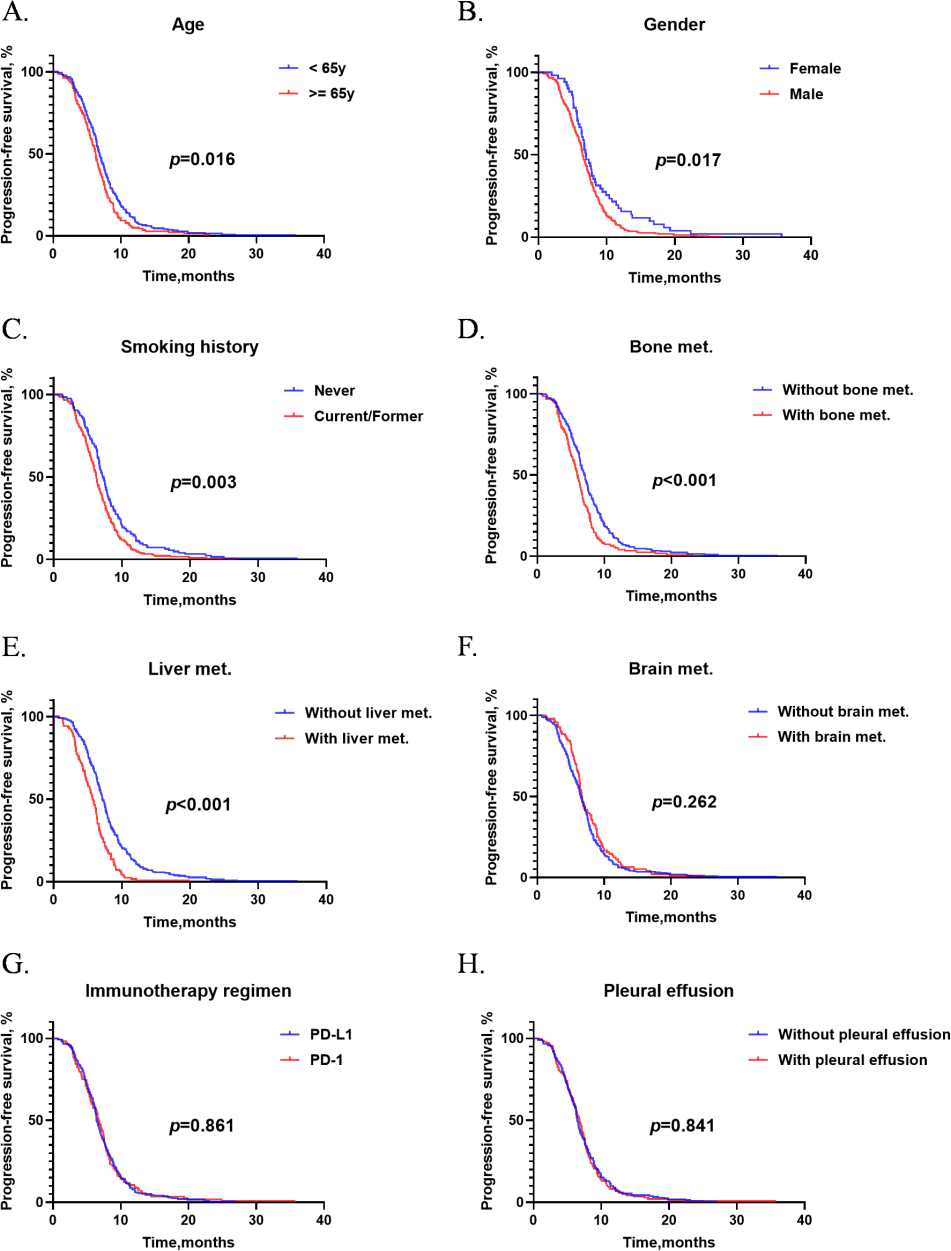
The subgroup analysis of PFS during first-line therapy. **(A)** Subgroup analysis of patients who received ICIs with different age. **(B)** Subgroup analysis of patients who received ICIs with different gender. **(C)** Subgroup analysis of patients who received ICIs with different smoking history. **(D)** Subgroup analysis of patients with and without liver metastases. **(E)** Subgroup analysis of patients with and without bone metastases. **(F)** Subgroup analysis of patients with and without brain metastases. **(G)** Subgroup analysis of patients with different ICIs; **(H)** Subgroup analysis of patients with and without pleural effusion. PFS, progression-free survival of first-line treatment; ICIs, immune checkpoint inhibitors; y, years; met., metastases.

Subgroup analysis in **Figure 3** showed that younger (*p* = 0.016, **Figure 3A**) and female (*p* = 0.017, **Figure 3B**) patients had longer first-line mPFS than older and male patients. Patients who have never smoked have a longer first-line PFS (*p* = 0.003, **Figure 3C**). Additionally, patients without bone metastasis showed better efficacy in first-line immunochemotherapy (*p* < 0.001, **Figure 3D**), as did those without liver metastasis (*p* < 0.001, **Figure 3E**). No significant differences of PFS were observed in the other subgroups including brain metastasis (*p* = 0.262, **Figure 3F**), immunotherapy regimen (*p* = 0.861, **Figure 3G**) and pleural effusion (*p* = 0.841, **Figure 3H**).

### Second-line therapy strategies

To further investigate the efficacy of continuous immunotherapy beyond disease progression after first-line immunochemotherapy, we excluded patients who showed primary immune resistance. Here, primary immune resistance refers to a PFS of less than 3 months following first-line therapy. Finally, a total of 206 patients were enrolled to compare the efficacy of second-line therapy. Among these 206 patients, 149 (72.3%) patients received continuous immunotherapy beyond progression, including 122 cases with immunochemotherapy, and other 27 cases with anti-angiogenic therapy plus immunotherapy (11 cases) or plus immunochemotherapy (16 cases). A total of 57 (27.7%) patients received chemotherapy without immunotherapy as second-line therapy, and among them 17 cases received anti-angiogenic agents at the same time. The subsequent strategies administered for patients after progression on first-line therapy were showed in **Figure 1** and the baseline clinical characteristics of 206 patients were showed in **Supplementary Table 2**.

### Second-line therapy efficacy among different groups

**Figure 4:**
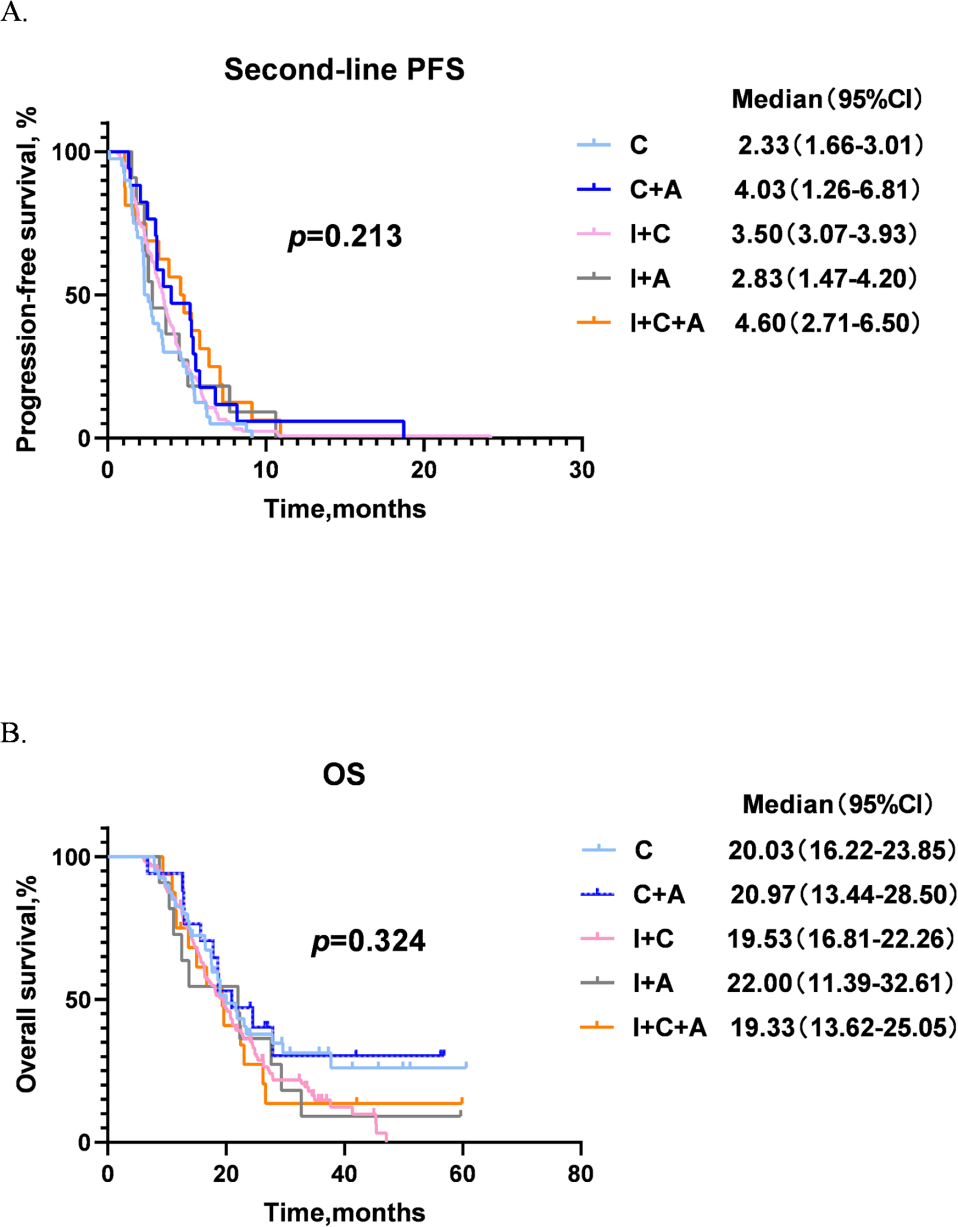
Second-line PFS and OS of patients among different second-line treatment groups. **(A)** Second-line PFS of different groups. **(B)** OS of different groups. PFS, progression-free survival; OS, overall survival; CI, confidence interval; C group, patients who received chemotherapy alone; C+A group, patients who received chemotherapy + anti-angiogenic therapy; I+C group, patients who received immunochemotherapy; I+A group, patients who received immunotherapy + anti-angiogenic therapy; I+C+A group, patients who received immunochemotherapy + anti-angiogenic therapy.

**Figure 5:**
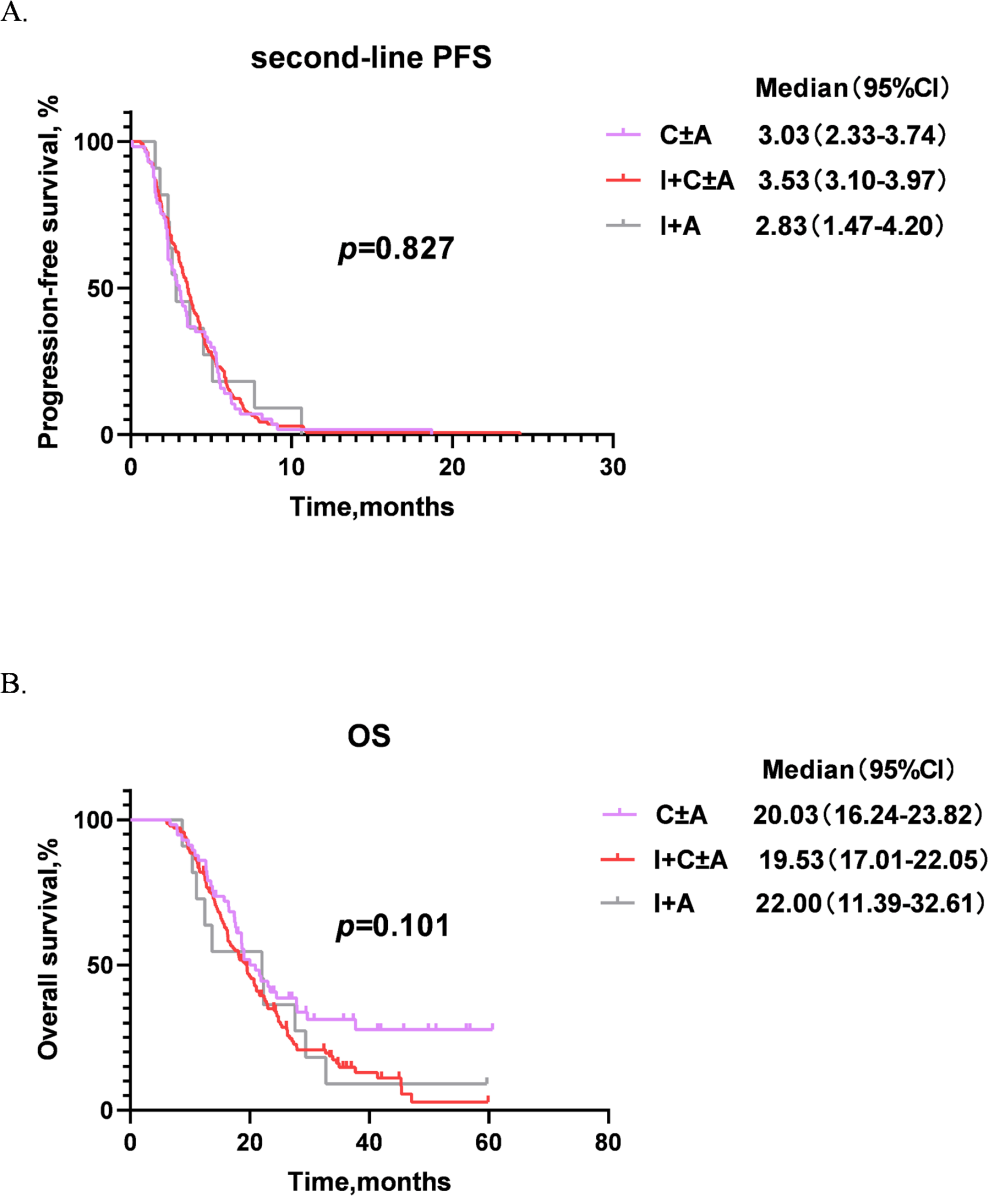
Second-line PFS and OS of patients among different second-line treatment groups. **(A)** Second-line PFS of different groups. **(B)** OS of different groups. PFS, progression-free survival; OS, overall survival; CI, confidence interval; C ± A group, patients who received chemotherapy ± anti-angiogenic therapy; I+C ± A group, patients who received immunochemotherapy ± anti-angiogenic therapy; I+A group, patients who received immunotherapy + anti-angiogenic therapy.

We categorized patients into five groups based on different second-line treatment regimens: chemotherapy alone (C, 40 cases), chemotherapy + anti-angiogenic therapy (C+A, 17 cases), immunochemotherapy (I+C, 122 cases), immunotherapy + anti-angiogenic therapy (I+A, 11 cases) and immunochemotherapy + anti-angiogenic therapy (I+C+A, 16 cases). The second-line PFS and OS among different groups are showed in **Figure 4**. Therein, I+C+A group obtained the longest second-line PFS of 4.60 months compared with other groups, next was the C+A group with a second-line PFS of 4.03 months. The second-line PFS of I+C group was longer than that of the C group, which were 3.50 months and 2.33 months respectively. The I+A group also showed poorer second-line PFS of 2.83 months compared with other groups (**Figure 4A**). As for OS, I+A group achieved best OS of 22.00 months, while I+C+A group showed the worst OS of 19.33 months. The other groups showed similar OS (20.97 months for C+A group, 20.03 months for C group and 19.53 months for I+C group) (**Figure 4B**). However, there were no statistical differences in second-line PFS and OS among the groups. Since chemotherapy and immunotherapy are the basic treatments, we further grouped C and C+A as C ± A, and grouped I+C and I+C+A as I+C ± A. It was found that I+C ± A showed the longest second-line PFS of 3.53 months (*p* = 0.827, **Figure 5A**), and I+A showed the longest OS of 22.00 months (*p* = 0.101, **Figure 5B**).

### Comparison between immunochemotherapy group and chemotherapy group

Since immunochemotherapy and chemotherapy were the main second-line treatment options, we subsequently compared the efficacy of immunochemotherapy (I+C, 122 cases) and chemotherapy (C, 40 cases) alone during second-line treatment. The baseline characteristics were well balanced according to the **Table 2**. The I+C group showed a longer second-line mPFS of 3.50 months compared with 2.33 months for chemotherapy group (*p* = 0.249, **Supplementary Figure 1**). Whereas, our data indicated that continuous immunotherapy did not show a longer mOS compared with chemotherapy without immunotherapy (19.53 vs. 20.03 months, *p* = 0.070, **Supplementary Figure 1**). There were no statistical differences in second-line PFS and OS between these two groups.

**Table 2:** Clinical characteristics of immunochemotherapy and chemotherapy alone as second-line treatment.

Characteristics	No. (%)		P value
	I+C group	C group	
Total	122 (100%)	40 (100%)	
Gender			
Male	105 (86.1%)	30 (75.0%)	0.141
Female	17 (13.9%)	10 (25.0%)	
Age, years			
Median age (range)	62 (42-79)	57 (38-76)	
<65	73 (59.8%)	31 (77.5%)	0.057
≥65	49 (40.2%)	9 (22.5%)	
ECOG PS			
0	46 (37.7%)	19 (47.5%)	0.722
1	73 (59.8%)	20 (50.0%)	
2	3 (2.5%)	1 (2.5%)	
Smoking history			
No	42 (34.4%)	17 (42.5%)	0.449
Yes	80 (65.6%)	23 (57.5%)	
Metastatic sites			
Liver	46 (37.7%)	13 (32.5%)	0.577
Bone	40 (32.8%)	15 (37.5%)	0.701
Brain	37 (30.3%)	9 (22.5%)	0.421
Cycles of Immunotherapy			
Median cycles(range)	7 (2-24)	6 (4-11)	
Chemotherapy regimen			
EC	74 (60.7%)	28 (70.0%)	0.190
EP	46 (37.7%)	10 (25.0%)	
Others	2 (1.6%)	2 (5.0%)	
Immunotherapy regimen			
Anti-PD-L1 antibody	85 (69.7%)	23 (57.5%)	0.178
Anti-PD-1 antibody	37 (30.3%)	17 (42.5%)	
Pleural effusion			
No	78 (63.9%)	23 (57.5%)	0.573
Yes	44 (36.1%)	17 (42.5%)	
Locoregional thoracic radiotherapy			
No	96 (78.7%)	27 (67.5%)	0.200
Yes	26 (21.3%)	13 (32.5%)	
Complications			
Hypertension	29 (23.8%)	9 (22.5%)	1.000
Diabetes	24 (19.7%)	7 (17.5%)	0.822
Coronary heart disease	10 (8.2%)	1 (2.5%)	0.296

No., number; ECOG PS, Eastern Cooperative Oncology Group Performance Status; EC, etoposide combined with carboplatin; EP, etoposide combined with cisplatin; PD-L1, programmed cell death ligand 1; PD-1, programmed cell death 1; C group, patients who received chemotherapy alone; I+C group, patients who received immunochemotherapy.

### The subgroup analysis of second-line PFS and OS between the immunochemotherapy group and chemotherapy group

**Figure 6:**
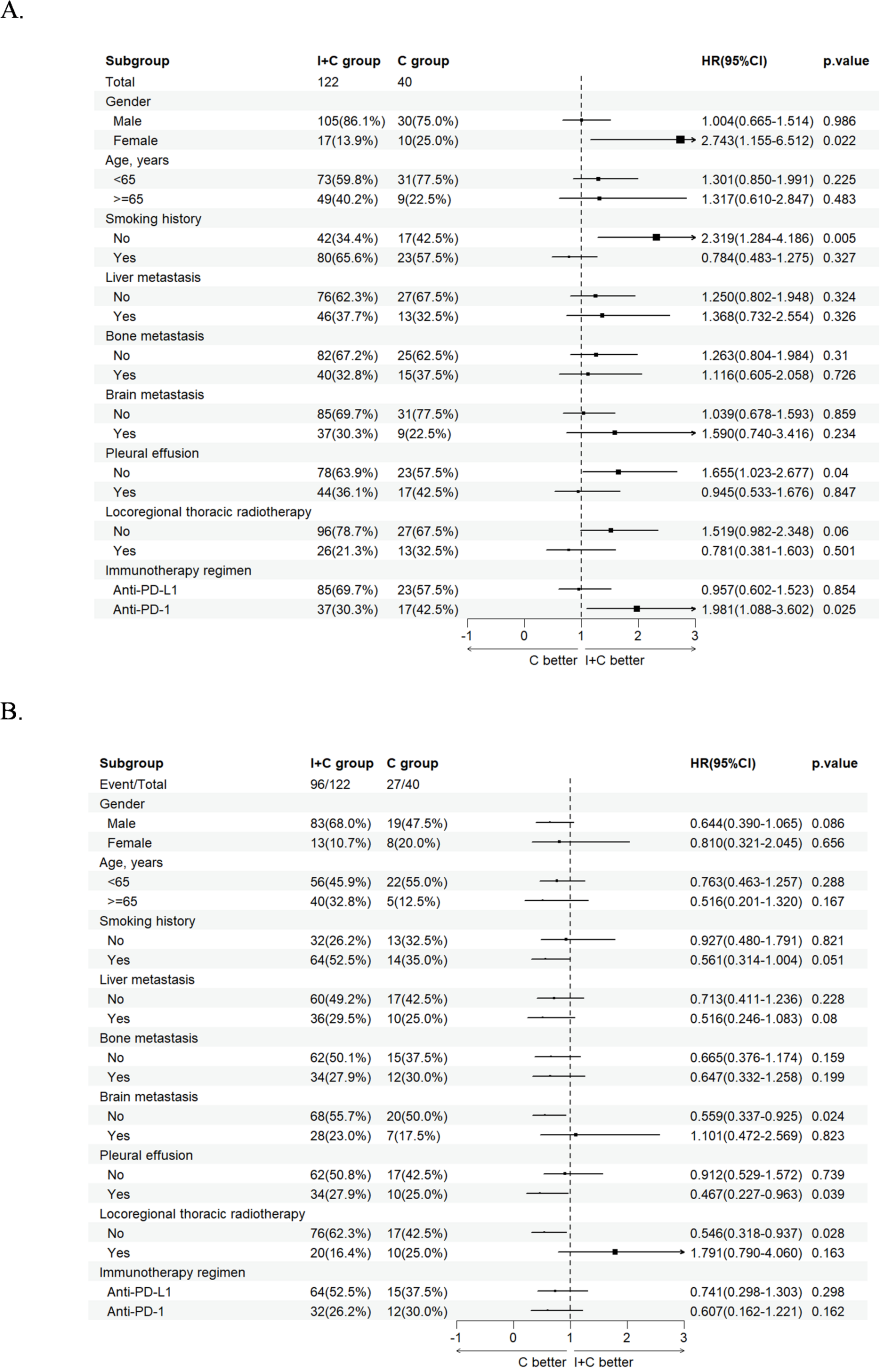
Forest plot of subgroup analysis comparing immunochemotherapy and chemotherapy group. **(A)** Forest plots of second-line PFS of I+C group and C group. **(B)** Forest plots of OS of I+C group and C group. PFS, progression-free survival; OS, overall survival; C group, patients who received chemotherapy alone; I+C group, patients who received immunochemotherapy; PD-L1, programmed cell death ligand 1; PD-1, programmed cell death 1; HR, hazard ratio; CI, confidence interval.

During the subgroup analysis of second-line PFS, we noted that the I+C group showed significant advantages over the C group in subgroups such as female patients, non-smokers, patients without pleural effusion, and patients treated with anti-PD-1 antibody, with statistical differences observed (**Figure 6A**). However, for OS, the C group demonstrated a significant advantage over I+C group in patients without brain metastasis, with pleural effusion and those did not receive locoregional thoracic radiotherapy (**Figure 6B**).

### The univariate and multivariate Cox regression analysis of second-line PFS and OS in immunochemotherapy and chemotherapy group

We used the univariate and multivariate Cox models to evaluate the effects of different variables on survival to determine which clinical characteristics are linked to the survival of ES-SCLC patients, which were summarized in **Tables 3**, **4**. The outcomes of the univariate analysis revealed that liver metastases, bone metastases and brain metastases were significantly associated with worse second-line PFS. Variables with a P-value between 0.05 and 0.10 were also incorporated into the subsequent multivariate Cox regression model. The multivariate analysis showed that liver metastases was a reliable predictive factor (**Table 3**). The univariate analysis also showed that liver metastases and bone metastases were significantly correlated with poorer OS. Upon conducting the multivariate analysis, liver metastases, bone metastases and second-line therapeutic regimen were identified as independent prognostic factors for OS, which was presented in **Table 4**.

**Table 3:** Univariate and multivariate Cox regression analysis of factors associated with second-line PFS.

Variable	Category	Univariate analysis		Multivariate analysis	
		HR (95%CI)	P value	HR (95%CI)	P value
Gender	Female vs. male	1.466(0.963-2.423)	**0.074**	1.241(0.809-1.905)	0.322
Age(years)	<65y vs. ≥65y	1.345(0.972-1.862)	**0.074**	1.288(0.928-1.789)	0.130
Smoking history	No vs. yes	1.183(0.854-1.641)	0.312	—	—
Liver metastases	No vs. yes	1.784(1.270-2.506)	**0.001**	1.585(1.115-2.255)	**0.010**
Bone metastases	No vs. yes	1.472(1.059-2.046)	**0.022**	1.252(0.890-1.763)	0.197
Brain metastases	No vs. yes	0.625(0.438-0.890)	**0.009**	0.774(0.532-1.127)	0.182
Pleural effusion	No vs. yes	0.844(0.612-1.164)	0.301	—	—
Locoregional thoracic radiotherapy	No vs. yes	0.970(0.675-1.392)	0.868	—	—
Therapeutic regimen(second-line)	Immunochemotherapy vs. Chemotherapy	1.234(0.861-1.768)	0.252	—	—

PFS, progression-free survival; y, years; HR, hazard ratio; CI, confidence interval. Values in bold indicate either statistical significance (P<0.05) or marginal significance (P=0.05-0.10).

**Table 4:** Univariate and multivariate Cox regression analysis of factors associated with OS.

Variable	Category	Univariate analysis		Multivariate analysis	
		HR (95%CI)	P value	HR (95%CI)	P value
Gender	Female vs. male	1.212(0.756-1.942)	0.424	—	—
Age(years)	<65y vs. ≥65y	1.230(0.852-1.776)	0.268	—	**—**
Smoking history	No vs. yes	1.182(0.817-1.709)	0.374	—	**—**
Liver metastases	No vs. yes	2.075(1.424-3.022)	**0.000**	2.039(1.387-2.997)	**0.000**
Bone metastases	No vs. yes	1.591(1.100-2.301)	**0.014**	1.507(1.033-2.197)	**0.033**
Brain metastases	No vs. yes	0.791(0.534-1.171)	0.241	—	**—**
Pleural effusion	No vs. yes	1.082(0.748-1.565)	0.677	—	**—**
Locoregional thoracic radiotherapy	No vs. yes	0.946(0.627-1.429)	0.793	—	**—**
Therapeutic regimen(second-line)	Immunochemotherapy vs. Chemotherapy	0.672(0.435-1.036)	**0.072**	0.595(0.384-0.923)	**0.020**

PFS, progression-free survival; y, years; HR, hazard ratio; CI, confidence interval. Values in bold indicate either statistical significance (P<0.05) or marginal significance (P=0.05-0.10).

### Safety

In terms of safety, we analyzed the AEs throughout the entire treatment process. The most common AEs of any grade in the immunochemotherapy group were anemia and fatigue, while the incidence of fatigue was higher in the chemotherapy group. For grade ≥3 AEs, we found that neutropenia predominated in both two groups. Furthermore, we analyzed the incidences of common irAEs in the two groups, noting that immune-related pneumonia was the most common irAE. However, the difference between the rates of AEs (including irAEs) in the two groups were not statistically significant (**Table 5**).

**Table 5:** Adverse events in immunochemotherapy and chemotherapy group.

Event	I+C group, No. (%) (N=122)	C group, No. (%) (N=40)	P value	I+C group, No. (%) (N=122)	C group, No. (%) (N=40)	P value
	Any grade	Any grade		Grade≥3	Grade≥3	
Total	120 (98.4%)	40 (100.0%)		78 (63.9%)	28 (70.0%)	
Anemia	**106 (86.9%)**	32 (80.0%)	0.309	22 (18.0%)	9 (22.5%)	0.643
Leukopenia	95 (77.9%)	33 (82.5%)	0.657	34 (27.9%)	15 (37.5%)	0.321
Neutropenia	92 (75.4%)	**35 (87.5%)**	0.125	**55 (45.1%)**	**23 (57.5%)**	0.203
Thrombocytopenia	49 (40.2%)	15 (37.5%)	0.853	11 (9.0%)	7 (17.5%)	0.153
Aminotransferase increased	52 (42.6%)	16 (40.0%)	0.854	12 (9.8%)	5 (12.5%)	0.766
Creatinine increased	9 (9.6%)	2 (5.0%)	0.520	1(0.8%)	0	1.000
Fatigue	**100 (82.0%)**	**36 (90.0%)**	0.322	3 (2.5%)	2 (5.0%)	0.598
Nausea	75 (61.5%)	22 (55.0%)	0.577	7 (5.7%)	1 (2.5%)	0.681
Vomiting	64 (52.5%)	20 (50.0%)	0.856	6 (4.9%)	0	0.338
Diarrhea	22 (18.0%)	7 (17.5%)	1.000	3 (2.5%)	0	0.576
Constipation	9 (7.4%)	3 (7.5%)	1.000	0	0	_
Immune-related adverse events						
Pneumonia	**10 (8.2%)**	**5 (12.5%)**	0.529	**6 (4.9%)**	**2 (5.0%)**	1.000
Hypothyroidism	5 (4.1%)	2 (5.0%)	1.000	0	0	_
Hyperthyroidism	2 (1.6%)	0	1.000	0	0	_
Rash	1 (0.8%)	0	1.000	1 (0.8%)	0	1.000
Adrenal insufficiency	2 (1.6%)	1 (2.5%)	1.000	0	0	_

No., number; C group, patients who received chemotherapy alone; I+C group, patients who received immunochemotherapy. Values in bold indicate the percentages of the most common adverse events.

## Discussion

ICIs combined with etoposide-platinum is recommended as standard first-line therapy for ES-SCLC (21, 22). Several ICIs including atezolizumab (6), durvalumab (3), adebrelimab (11), tislelizumab (13), toripalimab (14), serplulimab (12) and benmelstobart plus anlotinib (18) has been approved for the first-line treatment of ES-SCLC in China. Other ICIs such as pembrolizumab (9), ipilimumab (10) and tremelimumab (8) were also investigated in the first-line treatment of ES-SCLC. Despite immunochemotherapy has shown OS benefits in ES-SCLC compared with chemotherapy (23), improving second-line PFS and OS remains an unmet need for this lethal malignancy. For first-line immunotherapy-based combination therapy, there is no conclusive conclusion on whether to continue immunotherapy or not in the second-line treatment.

Patients with relapsed SCLC have globally dismal prognosis. Retreatment with original or similar platinum-based chemotherapy in patients with sensitive relapse has been widely adopted as routine practice, mostly because of the lack of highly active alternative treatment options (24). In the past, oral or intravenous topotecan was the drug approved for second line SCLC. This was based mainly on the results from two randomized phase III trials in which topotecan showed extended OS compared with best supportive care (25), and the noninferiority result in symptom control compared with cyclophosphamide, doxorubicin, and vincristine in patients relapsing after 60 days from first-line chemotherapy (26). However, the ORRs of topotecan regimen did not typically exceed 25%, and the median OS only ranged between 6–9 months (27). Irinotecan, lurbinectedin, paclitaxel, temozolomide and gemcitabine are among other modestly active drugs as single agents in the relapse setting. They are commonly used in routine clinical practice in second or further lines of treatment because of a better tolerability profile and somehow comparable clinical activity with topotecan. Anlotinib is a tyrosine kinase inhibitor of vascular endothelial growth factor receptor-2 (VEGFR-2) which demonstrated anti-tumour effects in various cancers and has been approved for later-line treatment of SCLC in China (28). Up to now, second-line or further lines treatment options for relapsed ES-SCLC after first-line immunochemotherapy are still controversial with limited overall survival benefit. Whether cross-line ICIs will improve clinical outcome for relapsed ES-SCLC remains ambiguous.

Continuous immunotherapy beyond progression for malignancies remains controversial as some studies have shown opposite results. In non-small cell lung cancer, results from the OAK study (29) found that the median post-PD OS was 12.70 months in 168 patients with continuing atezolizumab treatment beyond progression, while 8.80 months in 94 patients switching to non-protocol therapy. EMPOWER-Lung 1 explored first-line cemiplimab monotherapy and continued cemiplimab beyond progression plus chemotherapy for advanced NSCLC and found that for 64 patients who had disease progression on single-agent cemiplimab, second-line therapy with cross-line cemiplimab resulted in an ORR of 31.3% and a median OS of 15.10 months, which was superior to historical OS of 8.40 months with second-line chemotherapy alone (30). However, Enomoto et al. showed no remarkable advantages relevant to continuation of nivolumab for advanced NSCLC patients (15.60 vs. 13.40 months, P = 0.400) (31). Xu et al. found there were no significant benefits associated with continuation of original ICIs for advanced NSCLC patients beyond first-line immunotherapy progression (32). For small cell lung cancer, previous study have shown that rechallenging of PD-(L)1 inhibitors may offer benefits, especially for the first-line immunochemotherapy subgroup or those who had a SD or PD response to initial immunochemotherapy (33). The study of Liu et al. explored the clinical pattern of immunotherapy resistance in ES-SCLC and found that cross-line immunotherapy rechallenge had a better prognosis (34). Another study from Japan suggested the effectiveness of continuous ICIs beyond progression in clinical practice for SCLC and the strategy provided a favorable prognosis in selected cases of SCLC (35). The IMfirst Study explored the role of atezolizumab treatment beyond progression in ES-SCLC, supporting the continuous use of atezolizumab therapy after first-line immunotherapy progression (36). Another study assessed second-line outcomes for patients with ES-SCLC following progression after initial etoposide/platinum plus immunotherapy and found the overall second-line mOS (n=111) was 5.80 months (95%CI: 4.60-6.40). The mOS of non-rechallenge (n=88) group was 5.00 months (95%CI: 4.10-6.40) and that of rechallenge (n=23) group was 6.20 months (95%CI: 5.10-9.40), without statistically significance (*p* = 0.180) (37). In our study, we compared the efficacy of immunochemotherapy with chemotherapy alone during second-line therapy. The immunochemotherapy group showed a longer second-line mPFS of 3.50 months compared with 2.33 months for chemotherapy group (*p* = 0.249). Moreover, continued immunotherapy did not improve the OS of patients (19.53 vs. 20.03 months, *p* = 0.070), which might be partially due to the influence of immunotherapy rechallenge during subsequent-line treatment in the chemotherapy group.

As for anti-angiogenic agents, our study suggests that immunochemotherapy plus anti-angiogenic agents revealed the longest second-line PFS of 4.60 months versus other groups. The mOS of immunochemotherapy plus anti-angiogenic agents group is the longest with 22.00 months. The potential explanation is the synergistic effect of anti-angiogenesis, immunotherapy and chemotherapy in the reprogramming of the tumor microenvironment. This concept has been explored in patients with advanced non-small-cell lung cancer (38, 39). IMpower150 study demonstrated that the combination of ICIs plus anti-VEGF and chemotherapy was associated with superior OS than the combination of anti-VEGF and chemotherapy in NSCLC (19.20 versus 14.70 months, respectively). Phase 3 trial ETER701 investigated the efficacy and safety of benmelstobart (a novel PD-L1 inhibitor) with anlotinib and standard chemotherapy in treatment-naive ES-SCLC and achieved a median OS of 19.30 months which was longer than the reported OS in previous randomized clinical trials. The results suggest that the addition of anti-angiogenesis therapy to immunochemotherapy may represent an efficacious approach. And also, studies have revealed the synergistic effects of anti-angiogenic agents with immunotherapy could reprogram tumor microenvironment from an immunosuppressive one to an immune permissive microenvironment, and thus could be an opportunity to overcome immunotherapy resistance (40). A large number of studies have demonstrated that the combination therapy of anti-angiogenic agents and immunotherapy has good clinical application prospects, providing a hopeful solution to improve outcomes of cancer patients (41). Our study implied the potential benefits of anti-angiogenic agents in second-line setting. Compared with chemotherapy and immunochemotherapy, anti-angiogenic combinations showed no statistically significant difference in survival benefits, but a trend of second-line PFS and OS benefits. Certainly, the sample sizes in the I+A and I+C+A subgroups were limited, and additional studies with larger cohorts are warranted to confirm these findings.

Although immunotherapy and anti-angiogenic therapy can improve efficacy, chemotherapy still plays a cornerstone role. The second-line mPFS of I+A group is 2.83 months, while C ± A group is 3.03 months, and I+C ± A group is 3.53 months, indicating the important role of chemotherapy in second-line treatment. The role of later line chemotherapy in SCLC has been widely verified and is tolerable as monotherapy or combined therapy (4). Our findings suggest that chemotherapy represents the backbone of therapeutic management of SCLC, and the addition of immunotherapy or immunotherapy + anti-angiogenesis to chemotherapy during second-line therapy may contribute to PFS and OS prolongation.

The status of liver metastasis (LM) has been evaluated as a predictive biomarker in patients receiving ICIs, suggesting patients with LM derived limited benefit from immunotherapy independent of other established biomarkers of response (42). Mechanisms underlying hepatic immune tolerance included ineffective immune synapses resulting in T cell anergy, regulatory T cell induction or effector T cell elimination (42). Hepatocellular carcinoma is associated with hypoxic tumor conditions, high VEGF expression, and increased angiogenesis, which can contribute to the induction of immunosuppressive immune-cell types (e.g., myeloid-derived suppressor cells and regulatory T cells) and the promotion of immune tolerance in the tumor microenvironment (43). It has been reported that liver metastases diminish immunotherapy efficacy systemically in both preclinical models and cancer patients (42). In the present study, patients with LM showed worse first-line PFS from immunochemotherapy. Moreover, the univariate and multivariate analysis also showed that liver metastases was independent prognostic factors for poorer second-line PFS and OS. The explanation for these findings may lie in the immunosuppressive microenvironment within LM, which undermined the efficacy of immunotherapy (44).

It has been known that the presence of bone metastasis (BoM) was a negative prognostic factor in lung cancer (45). Bone metastasis, especially the occurrence of skeletal-related events (SREs), significantly reduces OS and quality of life (QoL) in patients. The lower efficacy of immunotherapy treatments in BoM patients could be induced by the presence of a particular immunosuppressive bone metastasis microenvironment (46, 47). Several studies suggested that bone involvement might be a negative prognostic factor and the presence of BoM could be predictive of poor response to ICIs (47–50). The present study also found poorer first-line PFS in patients with bone metastases. During second-line therapy, bone metastases were also significantly associated with worse second-line PFS and OS.

In terms of safety and toxicity, continuous immunotherapy beyond progression was not associated with unexpected safety events and all adverse events were generally manageable as previously reported. The combination of ICI and chemotherapy caused more ≥grade 3 adverse effects as fatigue, nausea, vomiting and diarrhea.

The present study showed that continuous immunochemotherapy beyond progression in patients with ES-SCLC does not enhance overall survival, which could be influenced by subsequent lines of therapy that patients may have received. It is important to acknowledge that the current study has innate limitations. First, this was a retrospective study with a comparatively small sample size. Thus, the results should be interpreted cautiously and should be further verified in future prospective randomized and controlled study with large sample size. Second, treatment heterogeneity could not be avoided in retrospective analysis, including the local treatments such as radiotherapy, and later-line medical treatments. Patient outcomes, especially the OS, might be partly impacted by these factors. Third, limited post-therapy scanning was unavoidable in a retrospective study analyzing data from real-world oncology clinical practice. The timing of the patient’s follow-up imaging examination following treatment or insufficient scans performed could have an influence on the results, especially PFS. Fourth, adverse events were retrospectively assessed based on medical records and reported for the entire treatment duration, with no stratification according to distinct treatment phases. Finally, patient selection bias was also one of the important limitations in this study. To exclude various biases, further large prospective randomized studies are warranted to confirm the effectiveness of continuous ICIs beyond progression in ES-SCLC.

## Conclusions

Continuous immunotherapy beyond progression in extensive-stage small cell lung cancer shows the trend of prolonging second-line progression free survival, but does not improve the overall survival. Additionally, in the second-line treatment, chemotherapy remains an important cornerstone therapy and anti-angiogenic agent containing strategy may potentially improve survival.

## Data Availability

The data presented in the study are included in the article/**Supplementary Material**. Further inquiries can be directed to the corresponding authors.

## References

[1] Bray F Laversanne M Sung H Ferlay J Siegel RL Soerjomataram I . Global cancer statistics 2022: GLOBOCAN estimates of incidence and mortality worldwide for 36 cancers in 185 countries. CA: A Cancer J Clin. (2024) 74:229–63. doi: 10.3322/caac.21834, PMID: 38572751

[2] Rudin CM Brambilla E Faivre-Finn C Sage J . Small-cell lung cancer. Nat Rev Dis Primers. (2021) 7:3. doi: 10.1038/s41572-020-00235-0, PMID: 33446664 PMC8177722

[3] Paz-Ares L Dvorkin M Chen Y Reinmuth N Hotta K Trukhin D . Durvalumab plus platinum–etoposide versus platinum–etoposide in first-line treatment of extensive-stage small-cell lung cancer (CASPIAN): a randomised, controlled, open-label, phase 3 trial. Lancet. (2019) 394:1929–39. doi: 10.1016/s0140-6736(19)32222-6, PMID: 31590988

[4] Megyesfalvi Z Gay CM Popper H Pirker R Ostoros G Heeke S . Clinical insights into small cell lung cancer: Tumor heterogeneity, diagnosis, therapy, and future directions. CA: A Cancer J Clin. (2023) 73:620–52. doi: 10.3322/caac.21785, PMID: 37329269

[5] Yu Y Chen K Fan Y . Extensive-stage small-cell lung cancer: Current management and future directions. Int J Cancer. (2023) 152:2243–56. doi: 10.1002/ijc.34346, PMID: 36346100

[6] Horn L Mansfield AS Szczęsna A Havel L Krzakowski M Hochmair MJ . First-line atezolizumab plus chemotherapy in extensive-stage small-cell lung cancer. New Engl J Med. (2018) 379:2220–9. doi: 10.1056/NEJMoa1809064, PMID: 30280641

[7] Liu S Reck M Mansfield A Mok T Scherpereel A Reinmuth N . Updated overall survival and PD-L1 subgroup analysis of patients with extensive-stage small-cell lung cancer treated with atezolizumab, carboplatin, and etoposide (IMpower133). J Clin Oncol. (2021) 39:619–30. doi: 10.1200/jco.20.01055, PMID: 33439693 PMC8078320

[8] Goldman J Dvorkin M Chen Y Reinmuth N Hotta K Trukhin D . Durvalumab, with or without tremelimumab, plus platinum-etoposide versus platinum-etoposide alone in first-line treatment of extensive-stage small-cell lung cancer (CASPIAN): updated results from a randomised, controlled, open-label, phase 3 trial. Lancet Oncol. (2021) 22:51–65. doi: 10.1016/s1470-2045(20)30539-8, PMID: 33285097

[9] Rudin CM Awad MM Navarro A Gottfried M Peters S Csőszi T . Pembrolizumab or placebo plus etoposide and platinum as first-line therapy for extensive-stage small-cell lung cancer: randomized, double-blind, phase III KEYNOTE-604 study. J Clin Oncol. (2020) 38:2369–79. doi: 10.1200/jco.20.00793, PMID: 32468956 PMC7474472

[10] Reck M Luft A Szczesna A Havel L Kim SW Akerley W . Phase III randomized trial of ipilimumab plus etoposide and platinum versus placebo plus etoposide and platinum in extensive-stage small-cell lung cancer. J Clin Oncol. (2016) 34:3740–8. doi: 10.1200/jco.2016.67.6601, PMID: 27458307

[11] Wang J Zhou C Yao W Wang Q Min X Chen G . Adebrelimab or placebo plus carboplatin and etoposide as first-line treatment for extensive-stage small-cell lung cancer (CAPSTONE-1): a multicentre, randomised, double-blind, placebo-controlled, phase 3 trial. Lancet Oncol. (2022) 23:739–47. doi: 10.1016/s1470-2045(22)00224-8, PMID: 35576956

[12] Cheng Y Han L Wu L Chen J Sun H Wen G . Effect of first-line serplulimab vs placebo added to chemotherapy on survival in patients with extensive-stage small cell lung cancer: the ASTRUM-005 randomized clinical trial. JAMA. (2022) 328:1223–32. doi: 10.1001/jama.2022.16464, PMID: 36166026 PMC9516323

[13] Cheng Y Fan Y Zhao Y Huang D Li X Zhang P . First-line chemotherapy with or without tislelizumab for extensive-stage small cell lung cancer: RATIONALE-312 phase 3 study. J Thorac Oncol. (2023) 18:S46. doi: 10.1016/j.jtho.2023.09.027

[14] Cheng Y Zhang W Wu L Zhou C Wang D Xia B . Toripalimab Plus Chemotherapy as a First-Line Therapy for Extensive-Stage Small Cell Lung Cancer: The Phase 3 EXTENTORCH Randomized Clinical Trial. JAMA Oncol. (2025) 11:16–25. doi: 10.1001/jamaoncol.2024.5019, PMID: 39541202 PMC11565370

[15] Ohe Y Han B Nishio M Watanabe S Ren X Murakami S . BEAT-SC: A randomized phase III study of bevacizumab or placebo in combination with atezolizumab and platinum-based chemotherapy in patients with extensive-stage small cell lung cancer (ES-SCLC). J Clin Oncol JCO. (2024) 42:8001–1. doi: 10.1200/JCO.2024.42.16_suppl.8001

[16] Spigel DR Townley PM Waterhouse DM Fang L Adiguzel I Huang JE . Randomized phase II study of bevacizumab in combination with chemotherapy in previously untreated extensive-stage small-cell lung cancer: results from the SALUTE trial. J Clin Oncol. (2011) 29:2215–22. doi: 10.1200/JCO.2010.29.3423, PMID: 21502556

[17] Tiseo M Boni L Ambrosio F Camerini A Baldini E Cinieri S . Italian, multicenter, phase III, randomized study of cisplatin plus etoposide with or without bevacizumab as first-line treatment in extensive-disease small-cell lung cancer: the GOIRC-AIFA FARM6PMFJM trial. J Clin Oncol Off J Am Soc. (2017) 35:1281–7. doi: 10.1200/JCO.2016.69.4844, PMID: 28135143

[18] Cheng Y Chen J Zhang W Xie C Hu Q Zhou N . Benmelstobart, anlotinib and chemotherapy in extensive-stage small-cell lung cancer: a randomized phase 3 trial. Nat Med. (2024) 30:2967–76. doi: 10.1038/s41591-024-03132-1, PMID: 38992123 PMC11485241

[19] Escudier B Motzer RJ Sharma P Wagstaff J Plimack ER Hammers HJ . Treatment beyond progression in patients with advanced renal cell carcinoma treated with nivolumab in checkMate 025. Eur Urol. (2017) 72:368–76. doi: 10.1016/j.eururo.2017.03.037, PMID: 28410865

[20] Mazieres J Rittmeyer A Gadgeel S Hida T Gandara DR Cortinovis DL . Atezolizumab versus docetaxel in pretreated patients with NSCLC: final results from the randomized phase 2 POPLAR and phase 3 OAK clinical trials. J Thorac Oncol. (2021) 16:140–50. doi: 10.1016/j.jtho.2020.09.022, PMID: 33166718

[21] Lahiri A Maji A Potdar PD Singh N Parikh P Bisht B . Lung cancer immunotherapy: progress, pitfalls, and promises. Mol Cancer. (2023) 22:40. doi: 10.1186/s12943-023-01740-y, PMID: 36810079 PMC9942077

[22] Zhang T . Immune checkpoint inhibitors in extensive-stage small cell lung cancer. J Natl Cancer Center. (2022) 2:130–1. doi: 10.1016/j.jncc.2022.07.003, PMID: 39036449 PMC11256711

[23] Zhou T Zhang Z Luo F Zhao Y Hou X Liu T . Comparison of first-line treatments for patients with extensive-stage small cell lung cancer: A systematic review and network meta-analysis. JAMA Network Open. (2020) 3:e2015748. doi: 10.1001/jamanetworkopen.2020.15748, PMID: 33074323 PMC7573680

[24] Ganti AKP Loo BW Bassetti M Blakely C Chiang A D’Amico TA . Small cell lung cancer, version 2.2022, NCCN clinical practice guidelines in oncology. J Natl Compr Cancer Network: JNCCN. (2021) 19:1441–64. doi: 10.6004/jnccn.2021.0058, PMID: 34902832 PMC10203822

[25] O’Brien MER Ciuleanu T-E Tsekov H Shparyk Y Čučeviá B Juhasz G . Phase III trial comparing supportive care alone with supportive care with oral topotecan in patients with relapsed small-cell lung cancer. J Clin Oncol. (2006) 24:5441–7. doi: 10.1200/jco.2006.06.5821, PMID: 17135646

[26] von Pawel J Schiller JH Shepherd FA Fields SZ Kleisbauer JP Chrysson NG . Topotecan versus cyclophosphamide, doxorubicin, and vincristine for the treatment of recurrent small-cell lung cancer. J Clin Oncol. (1999) 17:658–67. doi: 10.1200/JCO.1999.17.2.658, PMID: 10080612

[27] Zugazagoitia J Paz-Ares L . Extensive-stage small-cell lung cancer: first-line and second-line treatment options. J Clin Oncol. (2022) 40:671–80. doi: 10.1200/JCO.21.01881, PMID: 34985925

[28] Cheng Y Wang Q Li K Shi J Liu Y Wu L . Anlotinib vs placebo as third- or further-line treatment for patients with small cell lung cancer: a randomised, double-blind, placebo-controlled Phase 2 study. Br J Cancer. (2021) 125:366–71. doi: 10.1038/s41416-021-01356-3, PMID: 34006926 PMC8329046

[29] Gandara DR von Pawel J Mazieres J Sullivan R Helland Å Han J-Y . Atezolizumab treatment beyond progression in advanced NSCLC: results from the randomized, phase III OAK study. J Thorac Oncol. (2018) 13:1906–18. doi: 10.1016/j.jtho.2018.08.2027, PMID: 30217492

[30] Özgüroğlu M Kilickap S Sezer A Gümüş M Bondarenko I Gogishvili M . First-line cemiplimab monotherapy and continued cemiplimab beyond progression plus chemotherapy for advanced non-small-cell lung cancer with PD-L1 50% or more (EMPOWER-Lung 1): 35-month follow-up from a mutlicentre, open-label, randomised, phase 3 trial. Lancet Oncol. (2023) 24:989–1001. doi: 10.1016/s1470-2045(23)00329-7, PMID: 37591293

[31] Enomoto T Tamiya A Matsumoto K Adachi Y Azuma K Inagaki Y . Nivolumab treatment beyond progressive disease in advanced non-small cell lung cancer. Clin Trans Oncol. (2020) 23:582–90. doi: 10.1007/s12094-020-02452-1, PMID: 32661824

[32] Xu M Hao Y Zeng X Si J Song Z . Immune checkpoint inhibitors beyond first-line progression with prior immunotherapy in patients with advanced non-small cell lung cancer. J Thorac Dis. (2023) 15:1648–57. doi: 10.21037/jtd-22-1611, PMID: 37197488 PMC10183556

[33] Li L Liu T Liu Q Mu S Tao H Yang X . Rechallenge of immunotherapy beyond progression in patients with extensive-stage small-cell lung cancer. Front Pharmacol. (2022) 13:967559. doi: 10.3389/fphar.2022.967559, PMID: 36147357 PMC9485935

[34] Liu L Liu T Wang X Wang J Wang J Yuan M . Patterns of treatment failure for PD-(L)1 refractory extensive-stage small cell lung cancer in continued PD-(L)1 treatment. Trans Oncol. (2023) 33:101687. doi: 10.1016/j.tranon.2023.101687, PMID: 37182510 PMC10206491

[35] Yamamoto K Ninomaru T Okada H Hirano K Shimada T Hata A . Continuous immunotherapy beyond progression in clinical practice for small cell lung cancer. Thorac Cancer. (2024) 15:1271–5. doi: 10.1111/1759-7714.15308, PMID: 38623812 PMC11128369

[36] García-Campelo R Dómine Gómez M de Castro J Moreno Vega A Ponce Aix S Arriola E . P2.14–04 treatment beyond progression with atezolizumab in extensive-stage SCLC: exploratory analysis from the IMfirst study. J Thorac Oncol. (2023) 18:S372. doi: 10.1016/j.jtho.2023.09.656

[37] Andreas V Faltys M Alexander M Rogers J Parakh S Bowyer S . Clinical outcomes to second-line treatment, after failing chemoimmunotherapy in ES-SCLC. ESMO Abstract 202P. (2024).

[38] Lu S Wu L Jian H Cheng Y Wang Q Fang J . Sintilimab plus chemotherapy for patients with EGFR-mutated non-squamous non-small-cell lung cancer with disease progression after EGFR tyrosine-kinase inhibitor therapy (ORIENT-31): second interim analysis from a double-blind, randomised, placebo-controlled, phase 3 trial. Lancet Respir Med. (2023) 11:624–36. doi: 10.1016/s2213-2600(23)00135-2, PMID: 37156249

[39] Socinski MA Jotte RM Cappuzzo F Orlandi F Stroyakovskiy D Nogami N . Atezolizumab for first-line treatment of metastatic nonsquamous NSCLC. New Engl J Med. (2018) 378:2288–301. doi: 10.1056/NEJMoa1716948, PMID: 29863955

[40] Tu J Liang H Li C Huang Y Wang Z Chen X . The application and research progress of anti-angiogenesis therapy in tumor immunotherapy. Front Immunol. (2023) 14:1198972. doi: 10.3389/fimmu.2023.1198972, PMID: 37334350 PMC10272381

[41] Hu H Chen Y Tan S Wu S Huang Y Fu S . The research progress of antiangiogenic therapy, immune therapy and tumor microenvironment. Front Immunol. (2022) 13 802846. doi: 10.3389/fimmu.2022.802846, PMID: 35281003 PMC8905241

[42] Yu J Green MD Li S Sun Y Journey SN Choi JE . Liver metastasis restrains immunotherapy efficacy via macrophage-mediated T cell elimination. Nat Med. (2021) 27:152–64. doi: 10.1038/s41591-020-1131-x, PMID: 33398162 PMC8095049

[43] Chiu DKC Xu IMJ Lai RKH Tse APW Wei LL Koh HY . Hypoxia induces myeloid-derived suppressor cell recruitment to hepatocellular carcinoma through chemokine (C-C motif) ligand 26. Hepatology. (2016) 64:797–813. doi: 10.1002/hep.28655, PMID: 27228567

[44] Horst A Neumann K Diehl L Tiegs GJC . Modulation of liver tolerance by conventional and nonconventional antigen-presenting cells and regulatory immune cells. Cell Mol Immunol. (2016) 13:277–92. doi: 10.1038/cmi.2015.112, PMID: 27041638 PMC4856800

[45] Xue M Ma L Zhang P Yang H Wang Z . New insights into non-small cell lung cancer bone metastasis: mechanisms and therapies. Int J Biol Sci. (2024) 20:5747–63. doi: 10.7150/ijbs.100960, PMID: 39494330 PMC11528464

[46] Del Conte A De Carlo E Bertoli E Stanzione B Revelant A Bertola M . Bone metastasis and immune checkpoint inhibitors in non-small cell lung cancer (NSCLC): microenvironment and possible clinical implications. Int J Mol Sci. (2022) 23:6832. doi: 10.3390/ijms23126832, PMID: 35743275 PMC9224636

[47] Wu Y Zhang J Zhou W Yuan Z Wang H . Prognostic factors in extensive-stage small cell lung cancer patients with organ-specific metastasis: unveiling commonalities and disparities. J Cancer Res Clin Oncol. (2024) 150:74. doi: 10.1007/s00432-024-05621-9, PMID: 38305793 PMC10837219

[48] Gong L Xu L Yuan Z Wang Z Zhao L Wang P . Clinical outcome for small cell lung cancer patients with bone metastases at the time of diagnosis. J Bone Oncol. (2019) 19:100265. doi: 10.1016/j.jbo.2019.100265, PMID: 31763163 PMC6859228

[49] Qin A Zhao S Miah A Wei L Patel S Johns A . Bone metastases, skeletal-related events, and survival in patients with metastatic non–small cell lung cancer treated with immune checkpoint inhibitors. J Natl Compr Cancer Network. (2021) 19:915–21. doi: 10.6004/jnccn.2020.7668, PMID: 33878726 PMC8752085

[50] Schmid S Diem S Li Q Krapf M Flatz L Leschka S . Organ-specific response to nivolumab in patients with non-small cell lung cancer (NSCLC). Cancer Immunol Immunother. (2018) 67:1825–32. doi: 10.1007/s00262-018-2239-4, PMID: 30171269 PMC11028265

