# Supplementary material for: Clinical outcomes and safety of continuous immunotherapy beyond progression in patients with extensive-stage small cell lung cancer: a retrospective real-world study
Source: Front Immunol. 2026 Feb 6;16:1681545. doi: 10.3389/fimmu.2025.1681545 (PMC12920424; doi:10.3389/fimmu.2025.1681545)
Supplement: Supplementary file 1 [file DataSheet1.docx]

| Supplementary Table 1. Efficacy of first-line immunochemotherapy. | |
| --- | --- |
|  | **No.** |
| CR | 3 |
| PR | 218 |
| SD | 126 |
| PD | 7 |
| ORR | 62.4% (95%CI: 57.4%-67.5%) |
| PFS |  |
| Median, months(95%CI) | 6.60 (95%CI: 6.28-6.92) |
| No., number; ORR, objective response rate; PFS, progression free survival. | |

| Supplementary Table 2. Baseline clinical characteristics of the 206 patients. | | |
| --- | --- | --- |
| Characteristics | | **No. (%)** |
| Gender | Male | 171 (83.0%) |
|  | Female | 35 (17.0%) |
| Age, years | Median age (range) | 60.5 (38-81) |
|  | <65 | 132 (64.1%) |
|  | ≥65 | 74 (35.9%) |
| ECOG PS | 0 | 82 (39.8%) |
|  | 1 | 117 (56.8%) |
|  | 2 | 7 (3.4%) |
| Smoking history | No | 81 (39.3%) |
|  | Yes | 125 (60.7%) |
| Metastatic sites | Liver | 74 (35.9%) |
|  | Bone | 72 (35.0%) |
|  | Brain | 60 (29.1%) |
| Cycles of Immunochemotherapy | Median cycles(range) | 7 (2-15) |
| Chemotherapy regimen | EC | 129 (62.6%) |
|  | EP | 72 (35.0%) |
|  | Others | 5 (2.4%) |
| Immunotherapy regimen | Anti-PD-L1 | 144 (69.9%) |
|  | Anti-PD-1 | 62 (30.1%) |
| Immunotherapy regimen | Durvalumab | 58 (28.2%) |
|  | Atezolizumab | 49 (23.8%) |
|  | Adebrelimab | 34 (16.5%) |
|  | Serplulimab | 42 (20.4%) |
|  | Others | 23 (11.2%) |
| Pleural effusion | No | 124 (60.2%) |
|  | Yes | 82 (39.8%) |
| Locoregional thoracic radiotherapy | No | 154 (74.8%) |
|  | Yes | 52 (25.2%) |
| Complications | Hypertension | 54 (26.2%) |
|  | Diabetes | 38 (18.4%) |
|  | Coronary heart disease | 18 (8.7%) |
| No, number; ECOG PS, Eastern Cooperative Oncology Group Performance Status; EC, etoposide combined with carboplatin; EP, etoposide combined with cisplatin; PD-L1, programmed cell death ligand 1; PD-1, programmed cell death 1. | | |

**Supplementary Figure 1.** Kaplan-Meier analysis of Second-line PFS and OS between immunochemotherapy and chemotherapy alone as second-line treatment.


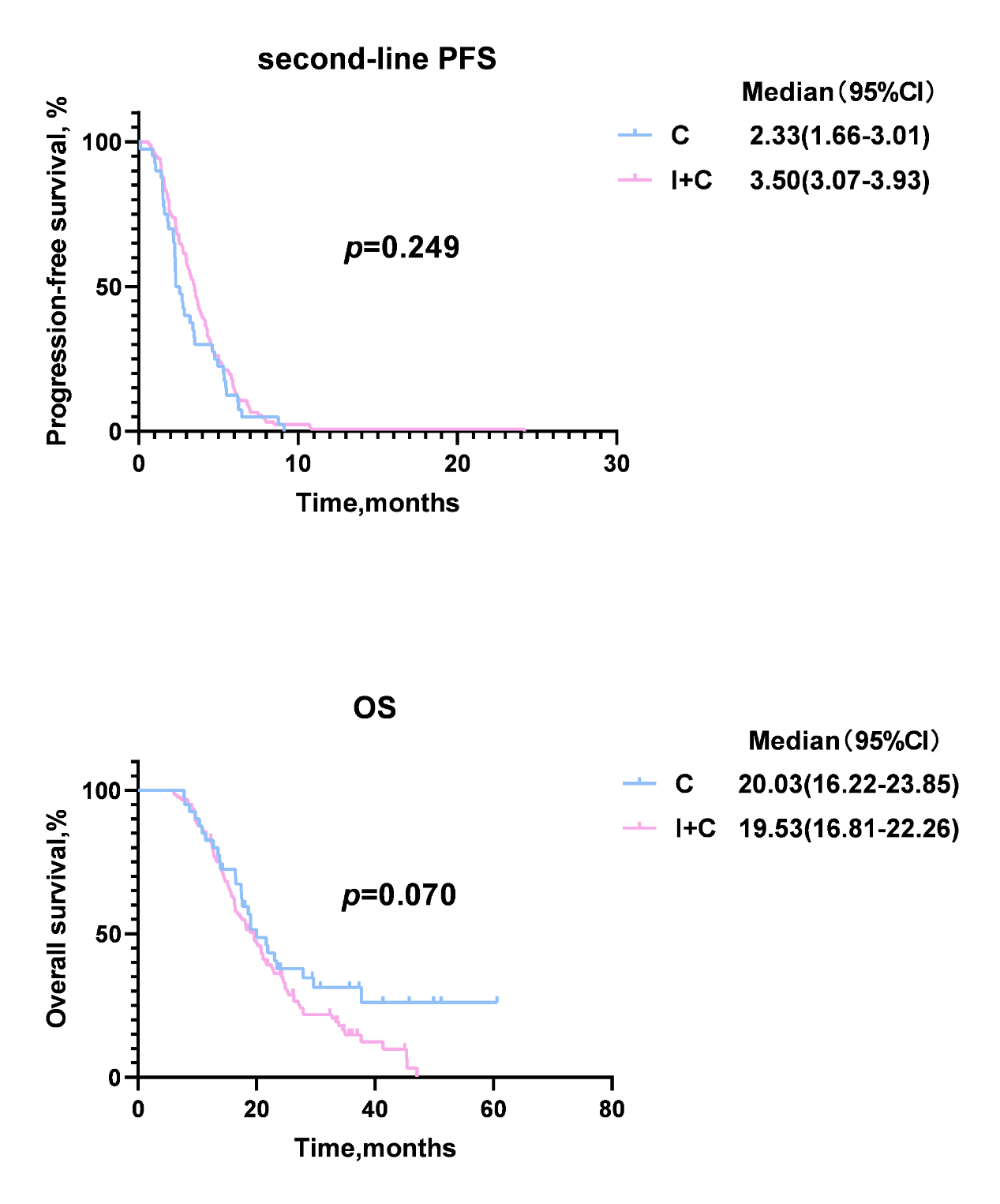


B.

A
